# Exact breakpoints of the *In(1)w ^m4^* rearrangement

**DOI:** 10.17912/micropub.biology.000608

**Published:** 2022-07-25

**Authors:** Alexander Solodovnikov, Sergey Lavrov

**Affiliations:** 1 Institute of Molecular Genetics of National Research Centre, Kurchatov Institute, Moscow, RUS

## Abstract

*
In(1)w ^m4^* has been known for decades as a classic example of a position effect variegation-causing rearrangement and has been mentioned in hundreds of publications. Nevertheless, its euchromatic breakpoint has not been localized with base-pair resolution. We performed nanopore sequencing of DNA from *In(1)w ^m4^* homozygous flies and determined the exact position of euchromatic (chrX:2767875) and heterochromatic breakpoints of the rearrangement. The heterochromatic breakpoint is located in an unlinked part of the genome in the region, enriched in TEs (transposable elements) fragments. A set of unique piRNAs could be detected in the region.

**Figure 1. In(1)w m4 inversion scheme:**
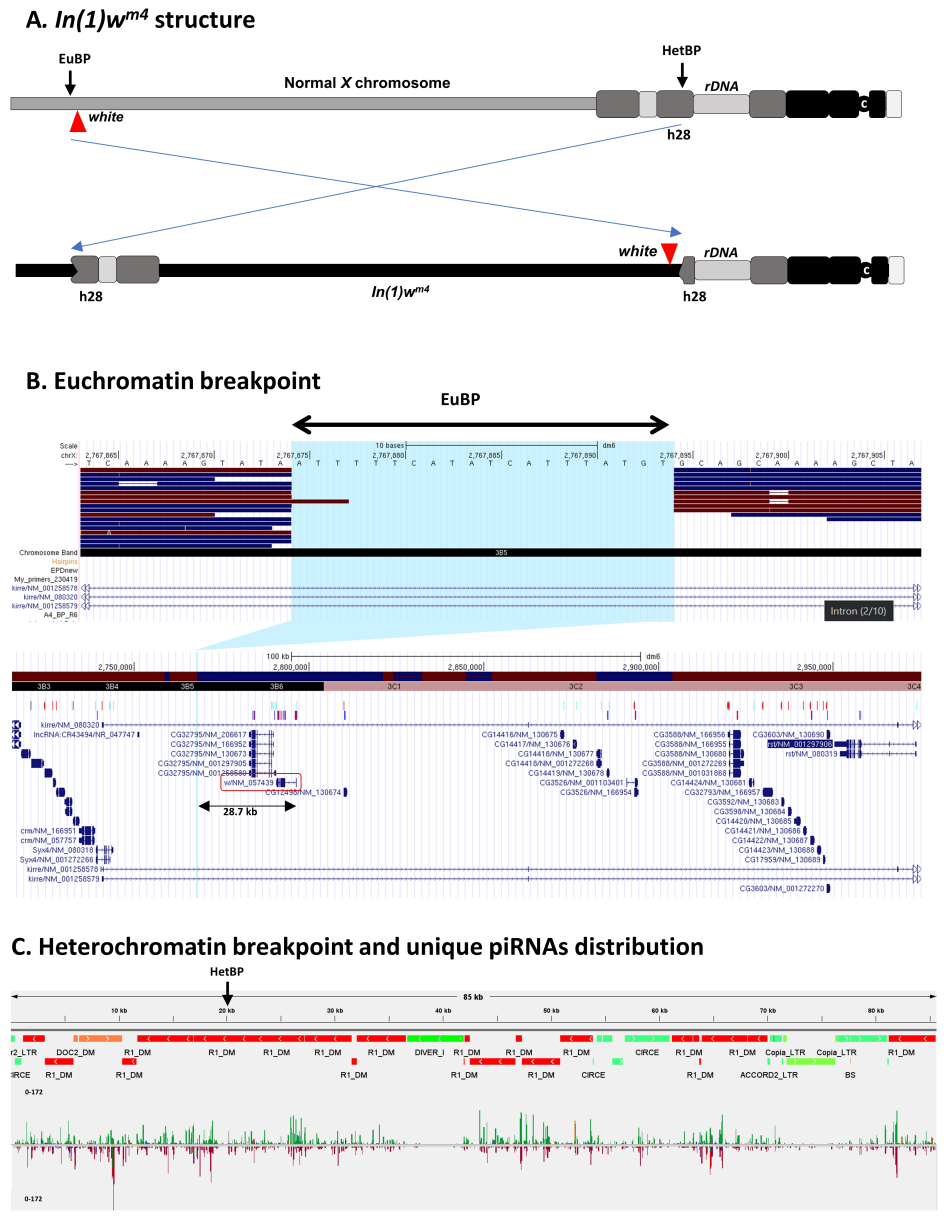
A. EuBP – euchromatin breakpoint, HetBP – heterochromatin breakpoint, c – centromere. The positions of the h28 heterochromatin block and the *rDNA* cluster are marked. Red triangles denote the position of the *white* gene. B. Mapping of long reads to the R6.22 *Drosophila* genome. The image is a collage of UCSC Genome Browser screenshots. The upper part is a close-up view of the euchromatic breakpoint and sequence reads. The lower part is an overview of the 200 kb genomic region containing the breakpoint. The *white* gene is outlined. The arrow shows the distance between the *white* TSS and the breakpoint. C. Heterochromatic breakpoint of *In(1)w ^m4^* inversion. The breakpoint in heterochromatin (HetBP) is in an R1 element in a region saturated with different types of mobile elements. The distribution of unique piRNAs in the region is shown below the TEs map (green – forward strand, red – reverse strand).

## Description

*In(1)w ^m4^* inversion represents the first described case of a heterochromatin position effect (Muller 1930). An inversion with the breakpoints near the *white* gene and in the heterochromatin of the left arm of chromosome X (Figure 1, A) results in a mosaic inactivation of the *white* gene visible in the eyes of adult flies. This rearrangement is a classic model of PEV and has been mentioned in a more than a hundred of publications. In particular, in (Vogel *et al.,* 2009), the chromatin changes in the case of *In(1)w ^m4^* -induces PEV were tracked using high-throughput methods (microarray hybridization and sequencing). In this study, the euchromatin breakpoint position of the inversion was determined with 1.5 kb accuracy (chrX:2766979–2768245, R6.22).

The heterochromatin breakpoint of *In(1)w ^m4^* was mapped to the h28 block of X chromosome heterochromatin distal to rDNA cluster, and the sequence immediately near the breakpoint corresponds to the R1 transposon (Appels *et al.,* 1982; Tartof *et al.,* 1984).

We decided to precisely map the positions of *In(1)w ^m4^* breakpoints in order to study the changes in chromatin organization immediately at the eu-heterochromatin border in the future. Nanopore sequencing was applied to look deep into the heterochromatin beyond the breakpoint position. Nanopore sequencing is characterized by a significantly higher number of errors than traditional NGS approaches, but has almost unlimited read length and, thus, extremely helpful in the assembly of repeat-rich regions of heterochromatin.

The results of our sequence analysis are presented in Figure 1. The gap in reads coverage was found in the genomic region chrX:2766979–2768245, which, as it was shown previously (Vogel *et al.,* 2009), contains the *In(1)w ^m4^* breakpoint. The position of the gap is chrX:2767875–2767894 according to the R6.22 release of the *D. melanogaster* genome (Figure 1, B). We used BLASTN to extract reads containing sequences immediately near this gap and investigate their composition. All these reads contained euchromatic sequences fused to the sequence of *Drosophila melanogaster* type I transposable element R1. The breakpoint in R1 corresponds to position 2200 in the GenBank X51968.1 R1 sequence.

In summary, the position of the euchromatic breakpoint of *In(1)w ^m4^* is chrX:2767875–2767894, the inversion is accompanied by a deletion of 20 nucleotides of euchromatin, and the breakpoint is 28.7 kb distal to the *white* TSS (Figure 1, B).

To identify sequences around the *In(1)w ^m4^* breakpoint in heterochromatin, reads of maximal length containing the euchromatic regions closest to the breakpoint were extracted from the library of reads. Reads overlapping the breakpoint and containing ~80 kb of heterochromatin were detected. The structure of the heterochromatin region was analyzed using RepeatMasker. We found that the heterochromatic breakpoint is in a region composed of fragments of transposable elements of different types (LINE (mainly R1) and LTR-containing), different orientations and lengths.

This organization resembles *Drosophila* piRNA-producing loci. We checked if the region is enriched in unique piRNAs using the *D. melanogaster* subset of piRBase and found a set of unique piRNAs distributed evenly on the forward and reverse strands (Figure 1, C), indicating a bidirectional piRNA production. The widely accepted trap model assumes production of piRNAs from TEs inserted into piRNA clusters and subsequent silencing of other copies of these elements in the genome (Bergman, Quesneville *et al.* , 2006, Duc, Yoth *et al.,* 2019, Ozata, Gainetdinov *et al.,* 2019). Remarkably, the insertion of *I* -element in the h28 heterochromatic region leads to decreased reactivity in the *I-R* hybrid dysgenesis system, thus pointing to the suppression of *I* -element transpositions (Dimitri *et al.,* 2005).

The heterochromatin sequences, located immediately near the PEV-inducing breakpoint, were identified in only a few cases. Extended blocks of simple satellites were identified in *In(1LR)pn2a* (Tolchkov, Kramerova *et al.,* 1997), *In(2)A4* (Abramov, Shatskikh *et al.,* 2016) and in *brown ^Dominant^* (Csink and Henikoff 1996). In the case of *In(1)w ^m4^* , the heterochromatin is of another type, it is a long region composed of rearranged TEs. It appears that PEV could be induced by structurally different types of heterochromatic sequences.

In contrast to simple satellites, there are numerous unique sequences in the heterochromatin region where the *In(1)w ^m4^* breakpoint is located. This gives the possibility to track the propagation of PEV-induced chromatin alterations into the heterochromatin using ChIP-Seq.

## Methods

*DNA isolation*

DNA from *In(1)w ^m4^ /In(1)w ^m4^* adult females (Bloomington stock 6209) was extracted using a proteinase K treatment – phenol-chloroform extraction – ethanol precipitation protocol. 100 flies were disrupted in 1 ml of TET buffer (100 mM Tris рН 8.5, 50 mM EDTA, 0.2% Triton Х-100) on ice using a Potter homogenizer. Sodium sarcosinate was added to 1.5% and proteinase K to 100 μg/μl, and the suspension was gently mixed and incubated for 1 h at 50 °C. The mixture was extracted twice with phenol pH 8.0, once with the phenol-chloroform mix, and once with chloroform in a Hula Mixer. The water phase after extraction was collected and sodium chloride was added to 100 mM. Then, 2 volumes of 96% EtOH were added and the tube was slowly rotated until HMW DNA formed a tangle. The DNA was washed once with 500 μl of 70% EtOH, dried briefly, and dissolved in 100 μl of MilliQ water. The DNA concentration was measured using a Qubit fluorimeter (dsDNA Broad Range kit). Liquid handling at every stage was performed gently and using cut-off pipette tips.

*MinIon sequencing*

The sequencing library was prepared from 1.5 μg of DNA according to the Ligation Sequencing Kit (SQK-LSK109) protocol from ONT. The prepared library was loaded into a MinIon R 9.4.1. flowcell and sequenced without basecalling until ~4 Gb of data was obtained.

*Data treatment*

A set of .fast5 files from the sequencing run was basecalled on a standalone GPU-enabled server with Guppy Version 3.5.2 using the dna_r9.4.1_450bps_hac profile. The resulting FASTQ files were loaded onto the local Galaxy server for further processing. Quality checks using Nanostat (https://github.com/wdecoster/nanostat) and Nanoplot (https://github.com/wdecoster/NanoPlot) tools in the Galaxy showed that ~2.4 Gb of reads (~20x genome coverage) with N50=14010 and Q>7 was obtained. Adapters were trimmed by Porechop (https://github.com/rrwick/Porechop) using default settings (reads with a middle adapter were split).

Processed reads were mapped to the R6.22 release of the *D. melanogaster* genome using Minimap2 software with the Oxford Nanopore read-to-reference mapping profile (minimap2 -x map-ont) (Li 2018). The resulting .bam file was visualized in the local UCSC Genome Browser (Figure 1, B). FASTQ files with the reads were also converted to FASTA using the FASTQ-to-FASTA converter in Galaxy (Blankenberg *et al.,* 2010).

The euchromatic breakpoint was identified as a gap in aligned reads in the 2 kb region where the *In(1)w ^m4^* breakpoint had been previously located (Vogel et al., 2009). Regions of 400 bp immediately upstream and downstream of the breakpoint (chrX:2767482–2767880 and chrX:2767881–2768281) were BLASTed against the sequencing reads in multifasta format to identify and extract reads overlapping the breakpoint. 32 reads containing euchromatic sequences near the presumptive breakpoint fused to heterochromatin sequences were identified. Two reads with the longest heterochromatic sequences to the left and right of the breakpoint were used to generate a single contiguous heterochromatic sequence. This sequence contains a region of heterochromatin ~85 kb in size, encompassing the *In(1)w ^m4^* breakpoint (Figure 1, C).

The structure of heterochromatin near the *In(1)w ^m4^* breakpoint was analyzed using the web application RepeatMasker (http://www.repeatmasker.org/cgi-bin/WEBRepeatMasker), which allows mapping of most types of *Drosophila* repeats. The output of the RepeatMasker was manually converted to a .bed file, color coded for different types of repeats and visualized in IGV (https://software.broadinstitute.org/software/igv/) (Figure 1, C). piRNA mapping was performed using the piRBase *Drosophila melanogaster* dataset ver. 23 (http://bigdata.ibp.ac.cn/piRBase/index.php). A custom *D. melanogaster* genome containing dm6 and the 85 kb heterochromatin region encompassing the *In(1)w ^m4^* breakpoint was constructed and then the piRBase dataset was mapped to this custom genome using Bowtie2 with -a option (the resulting .bam file in this case contains primary and secondary alignments). Then, the piRNAs having more than two matches were filtered out from .bam file and two bams for forward and reverse strands were produced. The results are visualized in IGV (Figure 1, C).

*Data availability*

The sequences encompassing the *In(1)w ^m4^* breakpoints and the 85 kb uninterrupted heterochromatin region are available in Extended Data. The MinIon dataset with *In(1)w ^m4^* reads is available from SRA (SRR17055722).

## Extended Data

Description: Mapping of transposable elements to the In(1)wm4 heterochromatiс breakpoint region. Resource Type: Model. DOI: 10.22002/D1.20230

Description: The sequence of the In(1)wm4 heterochromatin breakpoint region. Breakpoint position is 19978. . Resource Type: Dataset. DOI: 10.22002/D1.20231
